# Platelet Rich Plasma Combined with Arthroscopic Surgery Versus Arthroscopic Surgery Alone for the Treatment of Femoroacetabular Impingement Syndrome

**DOI:** 10.3390/jcm15135118

**Published:** 2026-07-01

**Authors:** Hao Ding, Zhongyao Li, Chunbao Li

**Affiliations:** The Fourth Medical Center, PLA General Hospital, Beijing 100048, China

**Keywords:** FAI, PRP, hip function, physical and mental health, minimal clinically important difference (MCID), patient-acceptable symptom state (PASS), clinical effectiveness

## Abstract

**Background/Objectives**: In patients with femoroacetabular impingement syndrome (FAI), the efficacy and safety of platelet-rich plasma (PRP) as an auxiliary treatment for femoroacetabular impingement syndrome (FAI) remain controversial. The study aimed to evaluate the safety and clinical effectiveness of arthroscopy combined with PRP compared with arthroscopy alone for the treatment of patients with FAI. **Methods:** In this retrospective study, patients who underwent hip arthroscopy for the treatment of FAI between January 2019 and January 2022 were included and divided into two groups: (1) arthroscopy group (A) and (2) arthroscopy combined with PRP group (AP). During the 2-year follow-up, Visual Analog Scale (VAS) pain scores, modified Harris Hip Score (mHHS), International Hip Outcome Tool-12 (iHOT-12), Hip Outcome Score-Activities of Daily Living (HOS-ADL), Hip Outcome Score-Sport-Specific (HOS-SSS), 12-Item Short Form Physical Composite Summary and Mental Composite Summary (SF-12 PCS and MCS), medical related expenses were recorded. Minimal clinically important difference (MCID) and patient-acceptable symptom state (PASS) comparison for mHHS, HOS-ADL, and HOS-SSS scores between the two groups. VAS, mHHS, iHOT-12, HOS-ADL, HOS-SSS score, and changing trends were described pre-operation and one month, six months, one year, and two years after the operation. **Results:** A total of 107 FAI patients were included; 55 patients were in Group A, 52 patients in Group AP. The overall mean ± SD was 39.1 ± 12.6 years (arthroscopy group, (A): 18 females, arthroscopy combined with PRP group, (AP): 13 females). There were no significant differences in the general data between the two groups (*p* > 0.05). Except for the auxiliary tools subscale of the mHHS (*p* > 0.05), both groups showed statistically significant improvements at one year (*p* < 0.01) and two years (*p* < 0.01) after surgery than pre-operation in VAS, mHHS, iHOT-12, HOS-ADL, and HOS-SSS. The scores after two years of treatment were further improved compared with one year (*p* < 0.01). There were no differences between the two groups at one year and two years postoperatively in VAS, mHHS, iHOT-12, HOS-ADL, and HOS-SSS scores (*p* > 0.05). No differences in the achievement of MCID and PASS for mHHS, HOS-ADL, and HOS-SSS (*p* > 0.05). A significant difference in SF-12 PCS and MCS was observed between the two groups preoperatively and two years after the operation (*p* < 0.01). The Group AP had significantly higher scores at two years (*p* < 0.01) after surgery than the Group A in the SF-12 MCS. There was no significant difference in SF-12 MCS distribution (>46.5, 36–46.5, ≤36) (χ^2^ = 0.198, *p* > 0.01). The AP group had significantly higher indirect and absenteeism expenses in the year of surgery than Group A (*p* < 0.01). **Conclusions:** For patients with FAI, compared with arthroscopy alone, arthroscopy combined with PRP can significantly improve SF-12 MCS two years after therapy; however, there was no significant improvement in clinical results, and indirect treatment costs were higher. Arthroscopy combined with PRP has no obvious advantage and cannot be recommended as the first choice.

## 1. Introduction

Femoroacetabular impingement (FAI) is a common cause of hip pain, characterized by pathological contact between the femur and acetabulum of the hip joint. This can lead to a cascade of intra-articular damage, including focal cartilage defects, cartilage delamination, chondrolabral separation, and labral tears [1]. Over time, this cumulative damage may predispose the hip to the development of osteoarthritis [2]. While hip arthroscopy has become the standard of care for treating symptomatic FAI [3,4], a subset of patients continues to experience persistent pain or suboptimal functional outcomes years after surgery. Furthermore, the chronic nature of the condition can also lead to significant psychological distress [5,6], and the clinical efficacy of surgery needs to be further improved.

Platelet-rich plasma (PRP) has emerged as an effective therapeutic option for a wide range of musculoskeletal diseases [7]. A growing body of evidence from numerous studies indicates that intra-articular PRP injections can significantly alleviate pain, improve physical function [8], and modulate local inflammatory responses [9]. These promising clinical outcomes have spurred growing interest in the application of PRP for treating various joint pathologies [8].

The efficacy of intra-articular PRP injections during hip arthroscopy remains debated. Some studies have reported no significant improvements in pain or functional outcomes with the addition of PRP [10,11], while others have demonstrated its significant clinical benefits [12,13]. This controversy is largely due to the lack of high-quality, conclusive evidence [14,15,16].

Therefore, the primary objective of this study was to investigate the efficacy of adjunctive PRP in hip arthroscopy for FAI. We performed a comprehensive comparison between patients treated with arthroscopy alone and those treated with arthroscopy combined with an intra-articular PRP. We hypothesized that while the addition of PRP may not lead to superior improvements in hip-specific function, it would be associated with greater benefits in patient-reported quality of life, particularly in the mental health domain.

## 2. Methods and Materials

### 2.1. Participants

**Figure 1:**
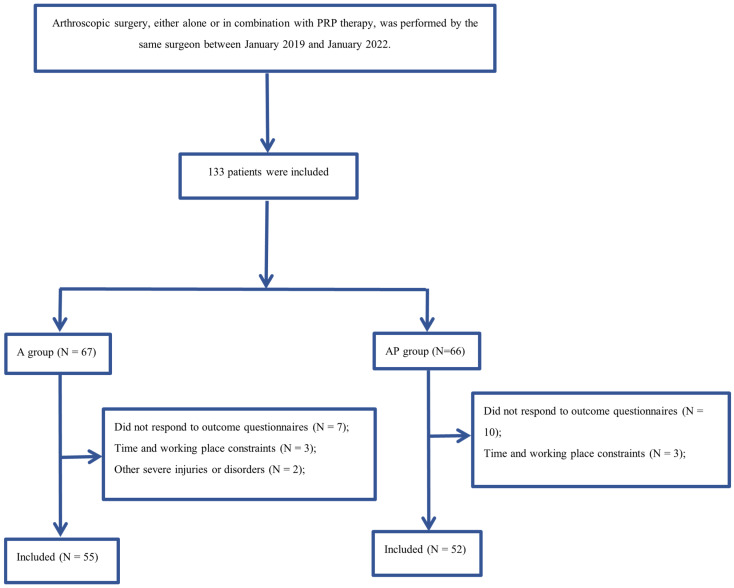
Flowchart of the inclusion process.

This retrospective cohort study was conducted at our institution. We identified patients who underwent hip arthroscopy for FAI between January 2019 and January 2022. The patient selection process is shown in Figure 1.

### 2.2. Inclusion and Exclusion Criteria

To be included in the study, patients had to meet the following criteria: (1) primary diagnosis of FAI requiring surgical intervention; (2) age ≥ 16 years at the time of surgery; and (3) all surgical procedures were performed by a single senior surgeon to minimize variability.

Patients were excluded if they had: (1) concomitant intra-articular pathologies treated during the same surgery, such as pigmented villonodular synovitis (PVNS) or synovial chondromatosis; (2) other sources of hip pain (e.g., proximal hamstring or abductor tendon pathology); or (3) evidence of moderate to severe pre-existing osteoarthritis (defined as a Tönnis grade ≥ 2).

### 2.3. Cohort Grouping

Based on the surgical treatment they received, the eligible patients were divided into two groups for comparison.

The Arthroscopy-alone (A) group: patients who underwent standard hip arthroscopy.

The Arthroscopy plus PRP (AP) group: patients who received an intra-articular PRP injection as an adjunct to arthroscopic surgery.

## 3. Surgical Technique

All surgical procedures were performed by a single, senior surgeon.

### 3.1. Standard Arthroscopic Procedure (Performed in Both Groups)

Following the induction of general anesthesia, the patients were placed in the supine position on a traction table. The operative hip was positioned in approximately 15° of abduction and 30° of internal rotation, while the contralateral hip was abducted to 45°. The hip joint was then distracted by approximately 1 cm using fluoroscopic guidance.

A standard anterolateral portal was established, and interportal capsulotomy was performed. Comprehensive diagnostic arthroscopy was then performed to assess all intra-articular structures, including the labrum, articular cartilage of the femoral head and acetabulum, and ligamentum teres. All identified pathologies were addressed accordingly.

Labral tears were treated with debridement, repair, or reconstruction based on tissue quality and tear characteristics.

Chondral lesions: Unstable chondral flaps were debrided to a stable margin. Microfracture was performed for full-thickness defects, if indicated.

Cam lesions: Femoral osteochondroplasty was performed to restore the normal head-neck offset.

Pincer lesions: Acetabuloplasty was performed for significant pincer-type impingement.

At the end of the procedure, the capsule was closed using multiple interrupted sutures.

### 3.2. Group-Specific Intervention

Arthroscopy-alone (A) Group: Patients in this group received no further intervention after the standard arthroscopic procedure.

Arthroscopy plus PRP (AP) Group: Seven days post-surgery, patients in this group received a single intra-articular injection of leukocyte-poor PRP. PRP was prepared from autologous venous blood according to the manufacturer’s protocol. Both groups adhered to the same rehabilitation protocol following treatment.

The fresh PRP samples were prepared at each weekly visit using a commercial product with single centrifugation at 1500× *g* for 5 min. This protocol yields a platelet concentration factor of 1 to 4 times more than whole blood values, with approximately 80% platelet recovery, and is leukocyte poor. The hip in the AP group received a single dose of PRP injection (5 mL). Due to some policy support, we offer free PRP treatment fees for patients.

## 4. Follow Up and Indicators

During the 2-year follow-up, all patients were recorded at baseline (pre-operation) and 2 years post-surgery. Periodic visits were conducted at 1 month, 6 months, and 1 year after the treatment to observe the changing trends. The 1 primary outcomes were 24-month change in overall average hip Modified Harris Hip Score (The mHHS includes questions that are assessed on a 0-to-100 scale in which 0 indicates poor hip-related function and 100 indicates excellent hip-related function).

### 4.1. Patient-Reported Outcome Measures (PROMs)

The following PROMs were administered:

*Pain:* Visual Analog Scale (VAS) for pain.

*Hip-specific Function and Symptoms:* Modified Harris Hip Score (mHHS). International Hip Outcome Tool-12 (iHOT-12). Hip Outcome Score (HOS), including Activities of Daily Living (HOS-ADL) and Sports-Specific Subscales (HOS-SSS).

*Health-Related Quality of Life (HRQoL):* The 12-Item Short Form Health Survey (SF-12), which yields the Physical Component Summary (PCS) and Mental Component Summary (MCS) scores.

*Cost Analysis:* Data on direct medical-related expenses were also collected for economic analysis.

### 4.2. Clinically Significant Outcomes

To interpret the clinical relevance of the observed changes, we used the concepts of MCID and PASS. Based on previously published studies [17,18], the following thresholds were applied:

MCID: Defined as an improvement of ≥8 points for the mHHS, ≥9 points for the HOS-ADL, and ≥6 points for the HOS-SSS.

PASS: Defined as a final outcome score of ≥74 for the mHHS, ≥87 for the HOS-ADL, and ≥75 for the HOS-SSS.

### 4.3. Health-Related Quality of Life and Mental Health Screening

Health-Related Quality of Life was assessed using the SF-12, a generic, self-administered questionnaire. The SF-12 yields two summary scores: the PCS and the MCS.

The SF-12 MCS is a well-validated measure of mental health and has demonstrated utility in screening for depression, with reported sensitivity and specificity of 0.83 and 0.87, respectively. In accordance with previous research [19], we used the established MCS cutoff scores for analysis:

An MCS score < 46.5 was used to identify patients with significant depressive symptoms.

An MCS score ≤ 36.38 was used to identify patients with potentially severe depressive symptoms.

## 5. Statistical Analysis

All statistical analyses were performed using SPSS software (Version 26.0). A two-sided *p*-value < 0.05 was considered statistically significant for all tests.

The normality of data distribution for continuous variables was assessed using the Kolmogorov-Smirnov (K-S) test.

### 5.1. Descriptive Statistics

Normally distributed continuous data were presented as mean ± standard deviation (SD).

Non-normally distributed continuous data were presented as median and interquartile range (IQR).

Categorical data were presented as frequencies and percentages (n, %).

### 5.2. Inferential Statistics

Baseline Comparisons between Groups (A vs. AP):

For normally distributed continuous variables, the independent samples *t*-test was used.

For non-normally distributed continuous variables, the Mann-Whitney U test was used.

For categorical variables, the chi-square (χ^2^) test or Fisher’s exact test was used as appropriate.

Longitudinal Analysis within Groups (Intra-group changes over time):

For normally distributed outcome variables measured at multiple time points (baseline, 1 month, 6 months, etc.), a repeated-measures analysis of variance (ANOVA) was used. The Student-Newman-Keuls (SNK) test was applied for post-hoc pairwise comparisons if a significant time effect was found.

For non-normally distributed or categorical outcome variables measured over time, generalized estimating equations (GEE) were used to model the longitudinal changes and compare trends between the two groups.

## 6. Results

### 6.1. Demographics

A total of 107 patients with FAI between January 2019 and January 2022 were included; 55 patients were in A group, 52 patients in AP group. The mean age was 39.1 ± 12.6 years (arthroscopy group, (A): 18 females, arthroscopy combined with PRP group, (AP): 13 females). There were no significant differences in general data between the two groups (*p* > 0.05) (Table 1). All patients signed the informed consent form for surgery.

### 6.2. VAS Score, mHHS Score, iHOT-12, HOS-ADL, HOS-SSS, and Subscale of mHHS

**Figure 2:**
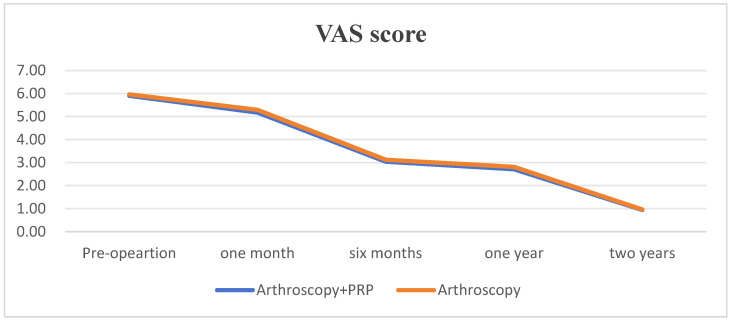
The changing trends of VAS score. PRP, platelet-rich plasma; VAS, Visual Analog Scale.

**Figure 3:**
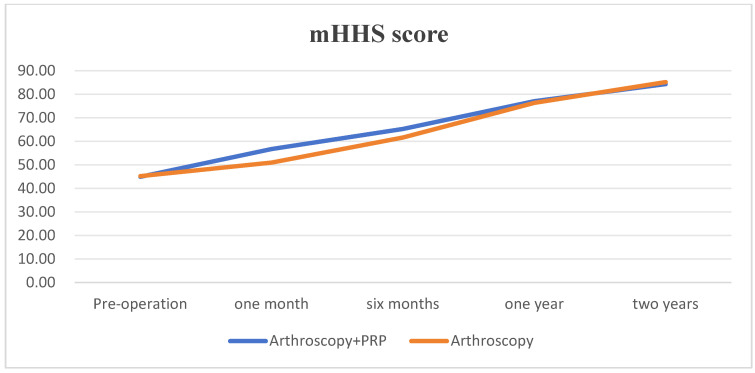
The changing trends of mHHS score. PRP, platelet−rich plasma; mHHS, modified Harris Hip Score.

**Figure 4:**
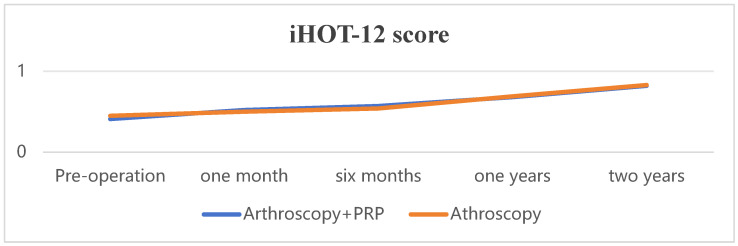
The changing trends of iHOT-12 score. PRP, platelet-rich plasma; iHOT-12, International Hip Outcome Tool-12.

**Figure 5:**
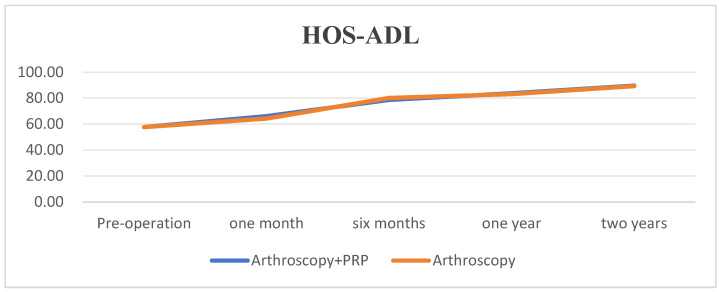
The changing trends of HOS-ADL score. PRP, platelet-rich plasma; HOS-ADL, hip outcome score-activities of Daily Living.

**Figure 6:**
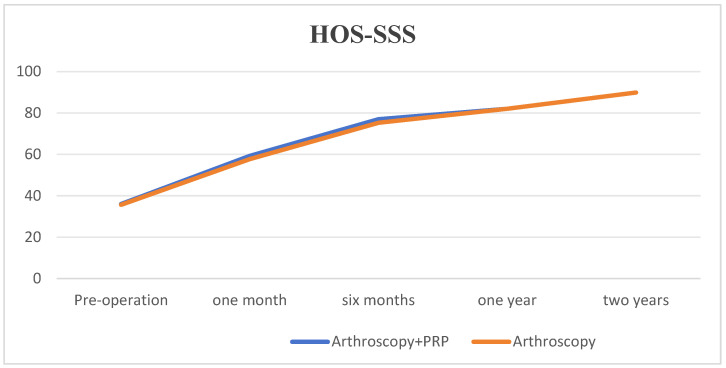
The changing trends of HOS-SSS score. PRP, platelet-rich plasma; HOS-SSS, hip outcome score-sport-specific.

Except for the auxiliary tools subscale of the mHHS (*p* > 0.05), the two groups had significantly higher scores at one year (*p* < 0.01) and two years (*p* < 0.01) after surgery than pre-operation in VAS, mHHS, iHOT-12, HOS-ADL, HOS-SSS, and subscale of mHHS. The two-year scores were further improved compared with one-year results (*p* < 0.01). There were no significant differences between the two groups at one year, two years after the operation in VAS, mHHS, iHOT-12, HOS-ADL, HOS-SSS, and subscale of mHHS (*p* > 0.05). The results are reported in Table 2, Supplementary Table S1 (Figure 2, Figure 3, Figure 4, Figure 5 and Figure 6).

### 6.3. MCID and PASS Comparison

No differences in the achievement of MCID and PASS for mHHS, HOS-ADL, HOS-SSS (*p* > 0.05) mHHS, HOS-ADL, HOS-SSS (*p* > 0.05), the results are reported in Table 3.

### 6.4. SF-12 Comparison

Two years after the treatment. A significant difference in SF-12 MCS was observed between the two groups at two years after the operation (*p* < 0.01). The AP group had a significantly higher score at two years (*p* < 0.01) after surgery than the A group in SF-12 MCS. There was no significant difference in SF-12 MCS distribution (>46.5, 36–46.5, ≤36) (χ^2^ = 0.198, *p* > 0.01). The results are reported in Table 4 and Table 5.

### 6.5. Medical Related Expenses

The AP group had significantly higher indirect costs and absenteeism expenses in the year of surgery than the A group (*p* < 0.01). There was no difference between the two groups in terms of economic losses caused by the low work efficiency and daily medical-related expenses at home (*p* > 0.05), as shown in Table 6.

## 7. Discussion

In this study, compared with arthroscopy alone, arthroscopy combined with PRP significantly improved SF-12 MCS two years after therapy; however, there was no significant improvement in clinical results, and indirect treatment costs were higher. It seems that arthroscopy combined with PRP has no obvious advantage and can not be recommended as the first choice.

Although arthroscopy is the standard of care for FAI [4,20], there is a theoretical rationale for using adjunctive PRP to enhance tissue healing, modulate inflammation, and improve clinical outcomes. Contrary to this expectation, we found that the addition of PRP did not lead to a statistically significant improvement in any hip-specific functional scores.

Several factors may explain this lack of efficacy. One possibility is the potential washout of PRP from the joint space. The interportal capsulotomy performed during surgery, even when repaired, may not provide a completely watertight seal, potentially leading to the leakage of the injected biologics [21].

A second, and perhaps more critical, factor is the suboptimal dosing and timing of the PRP application. The cellular response to PRP is known to be dose- and time-dependent. For example, a study [22] demonstrated that chondrocytes exhibit proliferation and metabolic activity at specific PRP concentrations and exposure durations. In our study, a single injection was administered one week after surgery. However, an exploratory observation of our data trends revealed that the AP group showed a slight, though non-significant, advantage over the control group around the 1-to-2-month postoperative mark.

This observation led us to hypothesize that the optimal therapeutic window for PRP’s effect may be later in the early postoperative period, when the initial inflammatory phase has subsided, and the proliferative healing phase is dominant. We speculate that administering a PRP injection (or a second booster injection) at 1 to 2 months post-surgery might yield a more pronounced clinical effect. This hypothesis warrants further investigation in future prospective studies designed to optimize the delivery protocol for PRP in the context of hip arthroscopy.

To assess the clinical relevance of our findings beyond statistical significance, we analyzed the MCID and the PASS. These metrics help determine whether observed changes are truly meaningful to patients. Our analysis revealed that at the two-year follow-up, a high percentage of patients in both the arthroscopy-alone and the PRP-adjuvant groups achieved the MCID and PASS thresholds for the mHHS, HOS-ADL, and HOS-SSS. Critically, there was no significant difference in the proportion of patients reaching these milestones between the two groups; this was consistent with the results that arthroscopy combined with PRP cannot further improve satisfaction [7,22].

However, the striking finding of our study was the differential effect on mental health. Despite the lack of difference in functional outcomes and satisfaction (as measured by PASS), the PRP group demonstrated a statistically significant and clinically meaningful higher SF-12 MCS score at two years. This suggests a dissociation between physical recovery and psychological well-being.

One possible interpretation is that while PASS reflects satisfaction with a specific functional state, the MCS score captures a broader sense of well-being, including confidence, hope, and resilience. The act of receiving an advanced biological therapy like PRP, irrespective of its direct physical effect, may have a powerful placebo or psychological-enhancement effect. Patients may feel they are receiving a more proactive and cutting-edge treatment, which could boost their confidence in recovery and positively influence their mental outlook. Therefore, while PRP did not improve functional satisfaction in our cohort, it appeared to enhance their mental health status through a pathway distinct from physical function.

FAI is a chronic condition, and the prolonged duration of symptoms before diagnosis and treatment can have a psychological impact. Studies have shown that even after arthroscopic surgery, a number of patients continue to suffer from depressive symptoms, which negatively affect their overall outcomes [23,24]. It was for this reason that we chose to assess mental health as a key secondary outcome.

Our study yielded a particularly intriguing finding: while the addition of PRP did not result in superior functional improvements (as measured by VAS, mHHS, and HOS), it was associated with a statistically significant and clinically meaningful improvement in mental health scores (SF-12 MCS). This finding suggests a potential dissociation between physical recovery and psychological well-being following PRP treatment for FAI.

We speculated that this improvement in mental health is not directly correlated with functional gains. Instead, it may be attributable to a powerful placebo or psychological-enhancement effect. This hypothesis is based on two considerations. First, patients in the PRP group were informed that they were receiving an advanced, autologous biological therapy intended to enhance healing. This knowledge itself—the belief that one is receiving a cutting-edge treatment—can foster a greater sense of hope, optimism, and self-confidence in the recovery process, thereby improving their mental outlook, it was a similarities with the results Lejla Pepic reported [25].

Second, this finding echoes the complex relationship between interventions and outcomes in FAI. For instance, while some studies, like that of Gruskay et al. [26], have found correlations between specific interventions and functional outcomes, our result aligns more closely with the principle that patient-reported outcomes can be influenced by factors beyond objective physical changes. The lack of a direct link between functional and mental improvement in our PRP group suggests that future studies should not only focus on physical metrics but also incorporate robust psychological assessments to fully capture the value of novel therapies.

In addition to clinical outcomes, we also conducted a preliminary analysis of treatment-related economic burdens. Our findings indicate that the AP group incurred significantly higher indirect costs, including greater income loss due to sick leave. However, there were no significant differences between the groups in terms of economic losses attributed to reduced work efficiency or daily medical-related expenses at home.

When weighing these economic considerations against the clinical outcomes, a clear picture emerges. The addition of PRP was associated with significantly higher costs but failed to provide superior benefits in any hip-specific functional measure or patient satisfaction metric (as indicated by PASS). The significant advantage in our study was an improvement in the mental component of quality of life.

Therefore, from a cost-effectiveness perspective, the routine use of adjunctive PRP in this setting appears unfavorable. We conclude that for the general FAI population, and particularly for patients with financial constraints, standard hip arthroscopy alone represents the more prudent and cost-effective treatment option.

### Limitation

Several limitations of this study warrant mention. Firstly, the limitation is its retrospective and non-randomized controlled study design, which is subject to inherent biases and precluded blinding of patients, clinicians, and outcome assessors. The lack of blinding may have introduced detection bias. Additionally, our two-year follow-up period may be insufficient to evaluate the long-term effects of the interventions. Future research, ideally in the form of a large-scale, prospective randomized controlled trial, is needed to confirm our findings and assess long-term outcomes. Furthermore, the observed dissociation between hip function and mental health scores highlights a complex relationship that requires more detailed investigation. For VAS score, the sample size is modest; a larger sample size is needed to confirm this.

## 8. Conclusions

For FAI patients, compared with arthroscopy alone, arthroscopy combined with PRP can significantly improve SF-12 MCS evaluation two years after the therapy; however, there was no significant improvement in clinical results, and indirect treatment costs was higher, it seems that arthroscopy combined with PRP has no obvious advantage and can be not recommended as the first choice.

## Figures and Tables

**Table 1:** Baseline Characteristics of the Study Population.

Groups	Number	Age	Sex (Male/Female)	BMI	Causes (Sports/Fall/Traffic)	Side (Left/Right)	Type (Cam-Type/Mixed-Type)	Time from Injury to Surgery	Cartilage Lesion Degree (I/II/III)	Number of Anchors
**A**	55	39.90 ± 12.20	37/18	23.2 ± 3.25	48/6/1	31/24	34/21	12.00 (5.00, 16.0)	32/21/2	3.34 ± 0.60
**AP**	52	39.60 ± 13.80	39/13	23.9 ± 3.72	46/5/1	30/22	30/22	12.0 (4.0, 25.0)	30/18/4	3.31 ± 0.70
**χ^2^/Z/t/Fiser value**		0.01	0.77	−0.82	0.33	0.01	0.19	−0.27	0.88	0.46
***p* value**		0.99	0.38	0.41	1.00	0.89	0.66	0.65	0.66	0.65

**Table 2:** Intergroup comparisons and intragroup comparisons of VAS, mHHS, iHOT-12, HOS-ADL, and HOS-SSS score M (Q1, Q3).

VAS score					
Groups	Pre-Operation	One Year	Two Years	χ^2^	*p* Value
**AP group**	6.00 (5.00, 7.00)	3.00 (2.00, 3.25) ^a^	1.00 (0.00, 2.00) ^ab^	152.86	<0.001
**A group**	5.00 (5.00, 7.00)	3.00 (2.00, 4.25) ^a^	1.00 (0.00, 2.00) ^ab^	89.16	<0.001
**Z value**	−0.70	−1.71	−1.87		
***p* value**	0.48	0.09	0.06		
**mHHS score**					
**Groups**	**Pre-operation**	**One year**	**Two years**	**χ^2^**	***p* value**
**AP group**	45.50 (37.00, 55.50)	80.00 (72.00, 84.00) ^a^	86.00 (84.00, 88.00) ^ab^	324.00	<0.001
**A group**	47.50 (39.75, 57.00)	77.00 (72.00, 82.50) ^a^	86.00 (82.00, 88.25) ^ab^	231.47	<0.001
**Z value**	−0.75	−0.60	−0.25		
***p* value**	0.46	0.55	0.80		
**iHOT-12 score**					
**Groups**	**Pre-operation**	**One year**	**Two years**	**χ^2^**	***p* value**
**AP group**	0.41 (0.23, 0.49)	0.68 (0.59, 0.73) ^a^	0.82 (0.70, 0.85) ^ab^	152.86	<0.001
**A group**	0.45 (0.26, 0.55)	0.69 (0.56, 0.80) ^a^	0.83 (0.69, 0.92) ^ab^	89.16	<0.001
**Z value**	−0.74	−0.62	−0.74		
***p* value**	0.46	0.54	0.46		
**HOS-ADL score**					
**Groups**	**Pre-operation**	**One year**	**Two years**	**χ^2^**	***p* value**
**AP group**	38.89 (0.28, 0.44)	83.33 (0.74, 0.92) ^a^	92.00 (0.83, 0.96) ^ab^	479.81	<0.001
**A group**	33.33 (0.22, 0.47)	90.00 (0.750, 0.92) ^a^	91.67 (0.83, 0.95) ^ab^	245.28	<0.001
**Z value**	−0.37	−0.68	−0.148		
***p* value**	0.71	0.50	0.88		
**HOS-SSS score**					
**Groups**	**Pre-operation**	**One year**	**Two years**	**χ^2^**	***p* value**
**AP group**	38.89 (0.28, 0.44)	83.33 (0.74, 0.92) ^a^	92.00 (0.83, 0.96) ^ab^	479.81	<0.001
**A group**	33.33 (0.22, 0.47)	90.00 (0.75, 0.92) ^a^	91.67 (0.83, 0.95) ^ab^	245.28	<0.001
**Z value**	−0.37	−0.68	−0.15		
***p* value**	0.71	0.50	0.88		

**Note:** AP, arthroscopy combined with PRP group; A, arthroscopy group; PRP, platelet-rich plasma; VAS, Visual Analog Scale; mHHS, modified Harris Hip Score; iHOT-12, International Hip Outcome Tool-12; HOS-ADL, hip outcome score-activities of Daily Living; HOS-SSS, hip outcome score-sport-specific. Comparison between pre-operation, ^a^ *p* < 0.01; comparison between one year after surgery, ^b^ *p* < 0.01.

**Table 3:** MCID, PASS comparison between the two groups [n (%)].

MCID				
Variables	AP Group (n = 52)	A Group (n = 55)	χ^2^ Value	*p* Value
**mHHS**	45	49	0.16	0.69
**HOS-ADL**	32	32	0.13	0.72
**HOS-SSS**	45	45	1.40	0.24
**PASS**				
**Variables**	**AP group (n = 52)**	**A group (n = 55)**	**χ^2^ value**	***p* value**
**mHHS**	47	48	0.67	0.41
**HOS-ADL**	41	41	0.28	0.60
**HOS-SSS**	41	44	1.66	0.20

**Note:** AP, arthroscopy combined with PRP group.

**Table 4:** SF-12 PCS and SF-12 MCS comparison between the two groups M (Q1, Q3).

SF-12 PCS					
Groups	Number	Pre-Operation	Two Years	χ^2^ Value	*p* Value
**AP group**	52	35.82 (33.18, 38.67)	43.72 (40.16, 48.54)	−5.96	<0.001
**A group**	55	35.02 (31.72, 36.31)	43.42 (41.67, 49.84)	−6.27	<0.001
**Z value**		−1.62	−0.75		
***p* value**		0.11	0.45		
**SF-12 MCS**					
**Groups**	**Number**	**Pre-operation**	**Two years**	**χ^2^ value**	***p* value**
**AP group**	52	34.82 (31.85, 36.88)	50.04 (47.20, 52.65)	−6.41	<0.001
**A group**	55	36.07 (33.28, 38.34)	48.43 (44.16, 50.38)	−6.44	<0.001
**Z value**		−1.39	−1.96		
***p* value**		0.164	<0.05		

Note: AP, arthroscopy combined with PRP group; SF-12 PCS, 12-Item short form physical composite summary; SF-12 MCS, 12-Item short form mental composite summary.

**Table 5:** SF-12 comparison between the two groups two years after the treatment [n (%)].

Variables	Group AP (n = 52)	Group A (n = 55)	χ^2^ Value	*p* Value
**>46.5**	40	41		
**36–46.5**	12	15	0.198	0.657
**≤36**	0	0		

Note: AP, arthroscopy combined with PRP group; SF-12, 12-Item Short Form Physical Composite.

**Table 6:** Expenses comparison between the two groups.

Groups (USD)	Absenteeism Expenses	Low Efficacy Expenses	Daily Medical Expenses	Indirect Cost/Year
**AP group**	1120.17 ± 323.62	303.10 ± 181.76	2270.34 ± 96.54	1736.42 ± 388.50
**A group**	1061.77 ± 261.87	300.87 ± 204.35	1973.43 ± 73.56	1634.83 ± 354.63
**t value**	1.13	0.07	1.84	1.84
***p* value**	0.03	0.95	0.07	0.04

**Note:** AP, arthroscopy combined with PRP group; **PRP**, platelet-rich plasma; **Absenteeism,** reduced productivity at work due to FAI treatment process, (*Total* income *for the* month)/(*the number of days* in the *month*) × period of leave (days), **Low efficacy,** low working efficacy due to FAI treatment process, (*Total* income *for the* month)/(*the number of days* in the *month*) × *work delay time* (days); **Daily medical expenses,** medical expenses of FAI surgery after discharge from the hospital; **Indirect cost,** sum of absenteeism, low efficacy and daily medical expenses. **USD:** United States Dollar.

## Data Availability

The original contributions presented in this study are included in the article/Supplementary Materials. Further inquiries can be directed to the corresponding author.

## Supplementary Materials

The following supporting information can be downloaded at: https://www.mdpi.com/article/10.3390/jcm15135118/s1, Table S1: Subscale intergroup and intragroup comparisons of mHHS M (Q1, Q3).
