# Supplementary material for: Platelet Rich Plasma Combined with Arthroscopic Surgery Versus Arthroscopic Surgery Alone for the Treatment of Femoroacetabular Impingement Syndrome
Source: J Clin Med. 2026 Jul 1;15(13):5118. doi: 10.3390/jcm15135118 (PMC13363635; doi:10.3390/jcm15135118)
Supplement: Supplementary file 1 [file jcm-15-05118-s001.zip › jcm-4170727-supplementary.pdf]

Supplementary Table S1. Subscale intergroup and intragroup comparisons of mHHS M (Q1,Q3)

Variables	Groups	Pre-operation	One year	Two years	χ^2 value	P value
**Pain**	AP group	20.00(10.00，20.00)	40(30，40)[a]	44(40.00，44.00)[ab]	406.73	<0.001
	A group	10.00(10.00，20.00)	40(30，40)[a]	44(40.00，44.00)[ab]	359.10	<0.001
	Z value	-0.30	-0.78	-0.06		
	P value	0.76	0.44	0.95		
**Gait**	AP group	24.00(18.00，27.00)	30.0(30.0，30.00) [a]	30.0(30.00，30.00) [ab]	59.03	<0.001
	A group	24.00(21.00，27.00)	30.0(28.50，30.00) [a]	30.0(30.00，33.00) [a]	43.01	<0.001
	Z value	-0.60	-0.46	-0.19		
	P value	0.55	0.64	0.85		
**Gait Limp**	AP group	8.00(5.00，11.00)	11(11.00，11.00)[a]	11.00(11.00，11.00)[ab]	25.57	<0.001
	A group	8.00(5.00，11.00)	11.00(11.00，11.00)[a]	11.00(11.00，11.00)[ab]	24.09	<0.001
	Z value	-0.09	-0.08	-1.14		
	P value	0.93	0.94	0.25		
**Assistant tool score**	AP group	11.00(11.00，11.00)	11(11.00，11.00)[a]	11.00(11.00，11.00) [ab]	5.18	0.08
	A group	11.00(11.00，11.00)	11(11.00，11.00) [a]	11.00(11.00，11.00) [ab]	5.03	0.06
	Z value	-0.33	-1.12	0.00		
	P value	0.74	0.26	1.00		
**Distance Walked**	AP group	5.00(2.00，5.00)	8.00(8，8.00)[a]	8.00(8.00，8.00)[ab]	137.27	<0.001
	A group	5.00(2.00，5.00)	8.00(8.00，8.00)[a]	8.00(8.00，11.00)[ab]	131.19	<0.001
	Z value	-0.54	-0.79	-0.51		
	P value	0.59	0.43	0.61		
**Functional Activities**	AP group	7.00(3.00，9.50)	12.0(10.00，14.00)[a]	12.0(12.00，14.00) [ab]	113.48	<0.001
	A group	8.00(4.00，10.00)	12.0(100.0，14.00) [a]	12.0(12.00，14.00) [ab]	97.57	<0.001
	Z value	-1.13	-0.34	-0.08		
	P value	0.26	0.74	0.94		
**Socks/Shoes**	AP group	2.00(2.00，4.00)	4.00(4.00，4.00)[a]	4.00(4.00，4.00)[ab]	50.38	<0.001
	A group	2.00(2.00，4.00)	4.00(4.00，4.00)[a]	4.00(4.00，4.00)[ab]	40.56	<0.001
	Z value	-1.20	-0.31	-0.46		
	P value	0.23	0.76	0.65		
**Sitting**	AP group	3.00(0.00，3.00)	3.00(3.00，5.00) [a]	3.00(3.00，5.00)[ab]	45.64	<0.001
	A group	3.00(0.00，5.00)	3.00(3.00，5.00)[a]	3(3.00，5.00)[ab]	39.48	<0.001
	Z value	-0.44	-0.13	-0.03		
	P value	0.66	0.90	0.97		

**Public transportation**	AP group	1.00(0.00，1.00)	1.00(1.00，1.00)[a]	1.00(1.00，1.00)[ab]	35.05	<0.001
	A group	1.00(0.00，1.00)	1.00(1.00，1.00)[a]	1.00(1.00，1.00)[ab]	21.74	<0.001
	Z value	-0.61	-0.62	0.00		
	P value	0.55	0.54	1.00		

Note: AP, arthroscopy combined with PRP group；FAI, femoro-acetabular impingement; PRP, platelet-rich plasma; mHHS, modified Harris Hip Score; comparison between pre-operation, [a] P<0. 01；comparison between one year after surgery,[b] P<0. 01.
